# Childhood immunization uptake determinants in Kinshasa, Democratic Republic of the Congo: ordered regressions to assess timely infant vaccines administered at birth and 6-weeks

**DOI:** 10.1186/s41256-023-00338-7

**Published:** 2023-12-06

**Authors:** Alix Boisson-Walsh, Peyton Thompson, Bruce Fried, Christopher Michael Shea, Patrick Ngimbi, Fidéle Lumande, Martine Tabala, Melchior Mwandagalirwa Kashamuka, Pélagie Babakazo, Marisa Elaine Domino, Marcel Yotebieng

**Affiliations:** 1 Department of Health Policy and Management, Gillings School of Global Public Health, The University of North Carolina, Chapel Hill, NC 27599 USA; 2 Division of Infectious Diseases, Department of Pediatrics, University of North Carolina, Chapel Hill, NC 27599 USA; 3 Ecole de Santé Publique de Kinshasa, Kinshasa, Democratic Republic of the Congo; 4 Center for Health Information and Research, College of Health Solutions, Arizona State University, Phoenix, AZ 85004 USA; 5 Division of General Internal Medicine, Department of Medicine, Albert Einstein College of Medicine, Bronx, NY 10461 USA

**Keywords:** Childhood vaccination, Immunization, Hepatitis B virus, Hepatitis B birth-dose vaccine, Tuberculosis, BCG, Vaccine distribution system, Expectant mothers, Health care workers, Intervention, Democratic Republic of the Congo

## Abstract

**Background:**

Despite global efforts to reduce preventable childhood illness by distributing infant vaccines, immunization coverage in sub-Saharan African settings remains low. Further, timely administration of vaccines at birth—tuberculosis (Bacille Calmette–Guérin [BCG]) and polio (OPV0)—remains inconsistent. As countries such as Democratic Republic of the Congo (DRC) prepare to add yet another birth-dose vaccine to their immunization schedule, this study aims to improve current and future birth-dose immunization coverage by understanding the determinants of infants receiving vaccinations within the national timeframe.

**Methods:**

The study used two ordered regression models to assess barriers to timely BCG and first round of the hepatitis B (HepB3) immunization series across multiple time points using the Andersen Behavioral Model to conceptualize determinants at various levels. The assessment leveraged survey data collected during a continuous quality improvement study (NCT03048669) conducted in 105 maternity centers throughout Kinshasa Province, DRC. The final sample included 2398 (BCG analysis) and 2268 (HepB3 analysis) women-infant dyads living with HIV.

**Results:**

Between 2016 and 2020, 1981 infants (82.6%) received the BCG vaccine, and 1551 (68.4%) received the first dose of HepB3 vaccine. Of those who received the BCG vaccine, 26.3%, 43.5%, and 12.8% received BCG within 24 h, between one and seven days, and between one and 14 weeks, respectively. Of infants who received the HepB3 vaccine, 22.4% received it within six weeks, and 46% between six and 14 weeks of life. Many factors were positively associated with BCG uptake, including higher maternal education, household wealth, higher facility general readiness score, and religious-affiliated facility ownership. The factors influencing HepB3 uptake included older maternal age, higher education level, household wealth, transport by taxi to a facility, higher facility general and immunization readiness scores, and religious-affiliated facility ownership.

**Conclusions:**

This study demonstrated that the study participants’ uptake of vaccines was consistent with the country average, but not in a timely manner. Various factors were associated with timely uptake of BCG and HepB3 vaccines. These findings suggest that investment to strengthen the vaccine delivery system might improve timely vaccine uptake and equity in vaccine coverage.

## Background

Childhood immunization against hepatitis B virus (HBV) was introduced to national vaccine schedules globally in the 1980s [1] but not instituted in the African continent until the early 2000s [1–3]. Accordingly, the prevalence of chronic HBV and its complications is still unacceptably high in sub-Saharan Africa (SSA). The World Health Organization (WHO) estimates that chronic hepatitis infects over 60 million individuals in SSA, [4] and the majority are unaware that they are carriers [5]. If infected at birth via mother-to-child transmission (MTCT), an infant has a 70–90% chance of developing chronic HBV infection and a 25% chance of mortality [6]. The ‘timely’ administration of HBV birth-dose vaccine (HepB-BD)—administered within the first 24 h after delivery and followed by additional vaccine doses—can prevent the overwhelming majority of MTCT cases [7–9]. Yet, in most of SSA, infants do not receive their first dose of the HBV vaccine series (HepB3) until six weeks, leaving them vulnerable to infection via MTCT. Timely receipt of HepB-BD is crucial in preventing MTCT. In a Cameroonian study of infants born to HBV surface antigen (HBsAg)-positive mothers, those who received HepB-BD within the first 24 h were found to have a 1.4% lower prevalence of HBsAg than those who received it 24–47 h after birth, and an 11.1% lower prevalence than those who received it 48–96 h after birth [10]. In addition, the risk of transmission among infants born to HBsAg-positive mothers was found to be eight times higher for those who received HepB-BD more than seven days after birth compared to those who received it within the first three days of life, [11] highlighting the importance of timely uptake.

Such high-burden countries as the Democratic Republic of the Congo (DRC) are preparing to add HepB-BD in their national immunization schedule for administration alongside other routine birth-dose (BD) vaccines–oral polio (OPV0) and tuberculosis (Bacille Calmette-Guérin [BCG]) [12]. However, effective implementation of BD vaccination is challenging in the SSA context. Of the 13 countries in the region that have already introduced HepB-BD into national policy, the reported coverage within 24h after birth is only 10% [13–15]. A study in the Gambia found that despite the availability of HepB-BD between 2004 and 2014, only 1.1% of infants received the vaccine within 24 h and only 5.4% by seven days after delivery [16]. These challenges are not unique to HepB-BD. A study in Kinshasa Province across health facilities highlighted significant challenges to the timely delivery of existing BD vaccines to newborns. Major determinants of the timely uptake of BCG and OPV0 included facility-level administration logistics, vaccine stockouts, and vaccine wariness and hesitation [17]. The current DRC national immunization schedule defines *timely delivery* of BCG and OPV0 BD vaccines as within the first seven days of life. Yet, ongoing challenges continue to impede timely BD delivery. Given these challenges, to introduce HepB-BD to the immunization schedule, facilities will require vaccine delivery strategies to proactively address barriers to administering three BD vaccines in an infant’s first 24 h of life. The DRC’s national immunization schedule currently awaits the introduction of HepB-BD; for the time being, the first round of HepB3 is provided at six weeks of age [18]. In such contexts, identifying determinants of timely administration of current BD vaccines—especially BCG, which is administered by an injectable method like HepB-BD, rather than orally like OPV0—can provide proximal and prospective insight into challenges to future timely HepB-BD uptake.

In the present study, we employ the Andersen Behavioral Health Model (BHM), used in previous immunization systems research, to categorize and understand the determinants of vaccine uptake [19]. The BHM addresses barriers to access and utilization at three levels: external environment, predisposing characteristics, and enabling resources, allowing us to investigate barriers to vaccine uptake at both the individual and facility levels. We must understand the determinants of infant receipt of timely vaccination to improve current and future BD immunization coverage. Therefore, we analyzed vital information about barriers and facilitators to the uptake of infant vaccines at multiple time points. Few studies assess barriers to timely first-round immunization at the individual and facility levels across multiple time points, and no study does so with the intent to improve future streamlined and timely uptake of HepB-BD.

## Methods

### Study design and setting

The assessment leveraged de-identified survey data collected during a continuous quality improvement (CQI) initiative study conducted at 105 maternity centers throughout the Kinshasa Province, DRC. The parent study was designed to assess the impact of continuous quality improvement interventions on long-term outcomes of antiretroviral therapy among pregnant and breastfeeding women. Therefore, eligible participants living with HIV were enrolled anytime during pregnancy, after delivery, or during well-child visits in postpartum at the select facilities.

Mother-infant pairs were excluded from this analysis if the mother or infant died during the study period, or if the child was aborted during the study period. The original study sample included 2875 expectant mothers enrolled in the cohort study from 2016 to 2020 during antenatal care (ANC) or postpartum visits. A face-to-face interview was conducted with all enrolled participants at registration and subsequent visits at the following time points: delivery (for pre-delivery enrolees), six weeks, six months, 12 months, 18–24 months, and 24+ months postpartum. The study staff collected information on the mother-infant pair at delivery. The infant's vaccination status was verified at each follow-up visit using vaccination cards and the vaccination registry in the clinic. Vaccine cards recorded the date of infant vaccines contemporaneously, so it was possible to calculate a precise number of days between the infant's birthday and vaccine date. Assessments about facility capacity and inventory were collected through interviews with facility managers and staff at each of the 105 facilities. The study selected the three clinics with the highest patient volume in each of the 35 health zones of the Kinshasa province. Mothers were eligible for inclusion in this analysis if they were enrolled in the parent study [20].

### Variables

**Fig. 1:**
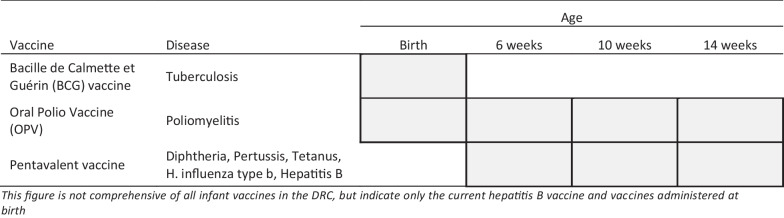
Current routine immunization schedule that begins at birth to 14 weeks, DRC 2022

The outcome measure of interest was infant vaccination status. We divided the outcome measure, BCG vaccine status into four categories: within 24 h of delivery, between one and seven days, or one week to 14 weeks; versus those who never received the BCG vaccine during the study period. The 14-week mark represents the moment of advised completion of an infant’s immunization schedule (Fig. 1). HBV vaccination status is divided into three categories: within 6 weeks, 6–14 weeks, and no uptake during the study period. The study team verified the vaccine status of all infants during verbal interviews with mothers and per review of immunization cards.

Covariates were selected based on existing literature about determinants of vaccine uptake at the community and facility levels [19, 21, 22]. The framework for variable selection was based on Andersen’s BHM to examine factors associated with vaccine utilization. The explanatory variables fell under the three BHM categories.

1. The external environment included the WHO General Service Availability and Readiness Assessment (SARA), [23] the WHO Immunization Services and Availability Assessment, facility type, ownership, and location—all assessed at the facility level. SARA was designed as a systematic survey to assess health facility service delivery, while the immunization service availability assessment was immunization service specific. Both scales consist of pre-selected tracer items within specific domains, which were carefully chosen and validated by service delivery experts. These domains represent essential treatment or care areas. Each item is binary, indicating the observed availability of the particular score on the day of the study team's facility visit. The general readiness scale is generated using four general service domains: basic amenities (6 items), basic equipment (6 items), standard precautions (9 items), and diagnostic capacity (8 items). The immunization readiness scale is composed of two domains: immunization service availability (6 items) and readiness (10 items). During the study, our staff observed the availability of tracer items in each facility. By calculating the mean composite score of the observed items within each domain, we derived a general readiness score and an immunization readiness score for each facility on a 10-point scale.
2. Predisposing characteristics included the infant’s birth order, mother’s age, her marital status, and her education level.
3. Enabling factors included the mother’s wealth index score and whether she traveled to the facility by foot or by transport. Table 1 includes more information about the covariates and their distributions.

**Table 1:** Explanatory variables for timely vaccine utilization, their operational definitions, and measures

Variable	Operational definition	Measure type
*Predisposing characteristics*		
Infant’s birth order	Number of infants birthed by the mother before the index infant	Continuous (children)
Mother’s age	Age at time of enrollment	Continuous (years)
Mother's education	Educational level of the mother at time of enrollment	Continuous (years)
Mother's marital status	Marital status of mother is captured at enrollment divided into two categories, not married (separated, divorced, widowed, never married) or married	Dichotomous 0 = not married; 1 = married
*Enabling factors*		
Household wealth index	Produced from existing variables (﻿household assets ownership, household characteristics and access to utilities) from enrollment questionnaires through factor analysis using Principal Component Analysis (PCA)	Categorical 1 = low wealth; 2 = mid-low wealth; 3 = mid-high wealth; 4 = high wealth
Transport to facility	Whether the mother traveled by foot or using transportation to the facility, captured at 6-week follow-up visit	Dichotomous 0 = walking; 1 = taxi or other
*External Environment*		
General Service Readiness Assessment	Produced from existing variables (basic amenities, basic equipment, infection prevention, and diagnostic capacity) from the facility inventory questionnaires through factor analysis using PCA	Continuous (points)
Immunization Services and Availability Assessment	Produced from existing variables (staff guidelines, immunization equipment, medicine and commodities) from the facility inventory questionnaires also through factor analysis using PCA	Continuous (points)
Facility type	Health center or reference hospital, captured in facility inventory questionnaire	Dichotomous 0 = reference hospital; 1 = health center
Facility Ownership	The gestational authority of the facility, captured in facility inventory questionnaire	Categorical 1 = public; 2 = religious-affiliation; 3 = private & other
Location	Urban versus rural/peri-urban status, captured in facility inventory questionnaire	Dichotomous 0 = rural/per-urban; 1 = urban

### Statistical analysis

We used multivariate ordered logistic regressions to run two versions of our analysis. Model I examined current BCG BD uptake, while Model II examined current HepB3 vaccine series uptake. With Model I, we examined determinants of current BD vaccines to anticipate barriers to future HepB-BD uptake. BCG uptake was the chosen proxy rather than OPV0 uptake because the injectable method of administration of the vaccine more closely approximated that of future HepB-BD than the orally administered polio vaccine. Model II measured determinants of the uptake of the first dose of HepB3, currently administered at six weeks of age in the DRC.

For model specifications, we used Akaike’s Information Criterion (AIC) to compare alternative functional forms and select the final model. Various functional forms of the continuous variables, including quadratic and categorical forms, were compared through an iterative approach. If a functional form was selected for one variable, it was retained for subsequent comparisons. Model fit was examined for both models, and the best functional forms with the minimum AIC were selected independently. Findings were reported in average marginal effects with delta-standard methods.

Both human error by vaccine staff conducting the interviews and loss-to-follow-up (LTFU) led to missing vaccine information across all time points for BCG status (missing, *n* = 239; LTFU, *n* = 248) and HepB3 status (missing, *n* = 369; LTFU, *n* = 281). LTFU was only measured among the participants who had not yet vaccinated their infants. We conducted two sets of sensitivity analyses for each outcome variable, BCG and HepB3, (a total of four analyses) to assess the robustness of the results of a full-case analysis against the alternative scenarios, including those with all missing or LTFU-specific information. The first set of analyses was defined by increasing the sample size to include participants with missing vaccine information across all time points as ‘not vaccinated’ or as a distinct category. The second set of analyses included only the LTFU-specific observations as ‘not vaccinated’ or as a distinct category. We found that a model with LTFU weights had directionality and magnitude in accordance with the majority of the models included in both sets of sensitivity analyses and therefore reported adjusted results accounting for LTFU.

## Results

### Sample characteristics

Between November 2016 and July 2020, the parent study enrolled 2,875 participants at 105 facilities. Of the enrolled participants, 56 mothers died, and 172 infants died or were aborted during the study period and were thus excluded from the analysis. A total of 239 mother-infant pairs did not provide BCG vaccine data, and 369 did not provide HepB3 vaccine information across any of the six follow-up visits after enrollment; these pairs were excluded from the analysis. The final sample with complete information included 2,398 (BCG analysis) and 2,268 (HepB3 analysis) women-infant dyads. Complete sample characteristics aggregated by the moment of the first infant vaccine can be found in Table 2.

**Table 2:** Characteristics of mother-infant pairs and facilities visited stratified by BCG vaccination status

	Overall		Vaccinated within 24 h		Vaccinated within 1 week		Vaccinated with 14 weeks		Not vaccinated	
	N = 2398		N = 630		N = 1044		N = 307		N = 417	
	No	(%)	No	(%)	No	(%)	No	(%)	No	(%)
*Mother's marital status*										
Married	1705	(71.10)	452	(71.75)	753	(72.13)	207	(67.43)	293	(70.26)
Not married	691	(28.82)	178	(28.25)	290	(27.78)	99	(32.25)	124	(29.74)
Missing	2	(0.08)	0	(0.00)	1	(0.10)	1	(0.33)	0	(0.00)
*Household wealth index*										
Low wealth	262	(11.13)	86	(13.65)	106	(10.15)	33	(10.75)	42	(10.07)
Mid-low wealth	883	(36.82)	200	(31.75)	371	(35.54)	121	(39.41)	191	(45.80)
Mid-high wealth	725	(30.23)	194	(30.79)	325	(31.13)	93	(30.29)	113	(27.10)
High wealth	523	(21.81)	150	(23.81)	242	(23.18)	60	(19.54)	71	(17.03)
*Transport*										
Walking	839	(34.99)	215	(34.13)	322	(33.89)	363	(34.77)	114	(37.13)
Taxi and other	1558	(64.97)	415	(65.87)	628	(66.11)	680	(65.13)	193	(62.87)
Missing	1	(0.04)	0	(0.00)	0	(0.00)	1	(0.10)	0	(0.00)
*Facility type*										
Health center	1399	(58.34)	366	(58.10)	622	(59.58)	174	(56.68)	237	(56.83)
Reference hospital	999	(41.66)	264	(41.90)	422	(40.42)	133	(43.32)	180	(43.17)
*Facility ownership*										
Public	770	(32.11)	208	(33.11)	318	(30.46)	86	(28.01)	158	(37.89)
Religious-affiliation	1,349	(56.26)	338	(53.67)	599	(57.38)	100	(58.63)	232	(55.64)
Private and other	279	(11.63)	84	(13.22)	127	(12.16)	41	(13.36)	27	(6.47)
*Location*										
Urban	1457	(60.76)	397	(63.02)	635	(60.82)	183	(59.61)	242	(58.03)
Rural/peri-urban	941	(39.24)	233	(36.98)	409	(39.18)	124	(40.39)	175	(41.97)

	M	(SD)	M	(SD)	M	(SD)	M	(SD)	M	(SD)
Infant's birth order	4.06	(2.20)	4.09	(2.16)	4.04	(2.16)	4.21	(2.38)	3.94	(2.21)
Mother's education	10.60	(2.99)	10.70	(3.02)	10.74	(2.98)	10.44	(3.06)	10.25	(2.91)
Mother's age	31.25	(6.10)	31.33	(6.06)	31.45	(6.04)	31.20	(6.19)	30.68	(6.21)
General readiness	6.77	(0.91)	6.73	(0.88)	6.84	(0.90)	6.83	(0.89)	6.63	(1.00)
General readiness##General readiness	46.72	(12.40)	46.07	(11.83)	47.59	(12.26)	47.46	(12.15)	44.98	(13.50)
Immunization readiness	5.82	(1.52)	5.88	(1.50)	5.76	(1.54)	5.89	(1.51)	5.86	(1.49)

Of the participants with vaccine data, 1981 (82.6%) received a BCG vaccine, and 1551 (68.4%) received a HepB3 vaccine. Of those who received the BCG vaccine, 630 (26.3%) of the participants received it within 24 h, 1044 (43.5%) received it between one and seven days, and 307 (12.8%) received it between one and 14 weeks. Of infants who received the first dose of HepB3 vaccine, 347 (22.4%) received it within six weeks, and 713 (46%) between six and 14 weeks of life.

In terms of predisposing characteristics, overall, the mean age of the mothers was 31 years old (S.D. = 6.099), and 1,705 (71.2%) mothers were married. Mothers had a median of four children (IQR: 2–5 children) and had 11 years of education (IQR: 9–12 years). For enabling factors, of the total eligible sample, 262 (11.1%) women fell within the lowest wealth quartile versus 523 (21.8%) who fell within the highest. 1558 (65%) of the women traveled to the facility by taxi (or other forms of transportation) rather than by foot. In terms of the external environment, overall, 1399 (58.3%) women sought care at health centers instead of hospitals, 1,349 (56.3%) women received care at a religiously-affiliated facility, and 1,457 (60.8%) women visited urban facilities. In addition, the median score for a mother’s facility where she sought care was 6.75 (IQR: 6.12–7.39) on the general readiness scale and 6.11 (IQR: 5–6.67) on the immunization readiness scale.

### Model I. Determinants of the uptake of BCG BD vaccine

Table 3 presents the results from the ordered logit analyses, which estimated how vaccine uptake at different time points was related to factors influencing the respondent at the predisposing, enabling, and external levels.

**Table 3:** Ordered logistic regression model results examining predisposing, enabling, and external predictors of infant vaccine uptake at different time points

	Model I BCG Vaccine (birth-dose)				Model II HepB3 Vaccine (6 weeks)		
*Variable*	Vaccinated within 24 h	Vaccinated within 1 week	Vaccinated with 14 weeks	Not vaccinated	Vaccinated within 6 weeks	Vaccinated within 14 weeks	Not vaccinated
*Infant's birth order*	0.0058	0.0018	− 0.00128	− 0.0064	0.0020	0.0010	− 0.0030
	(0.0040)	(0.0013)	(0.00087)	(0.0044)	(0.0036)	(0.0018)	(0.0054)
*Mother's age*	0.0018	0.00058	− 0.00040	− 0.0020	0.0021*	0.00106*	− 0.0031*
	(0.0014)	(0.00045)	(0.00031)	(0.0015)	(0.0012)	(0.00065)	(0.0019)
*Mother's education*	0.0048*	0.00153*	− 0.00100*	− 0.0052*	0.0077***	0.0040***	− 0.0117***
	(0.0027)	(0.00088)	(0.00060)	(0.0030)	(0.0024)	(0.0012)	(0.0035)
*Mother's marital status*							
Not married	0.0044	0.0014	− 0.0010	− 0.005	0.011	0.0053	− 0.016
	(0.017)	(0.0051)	(0.0037)	(0.018)	(0.015)	(0.0072)	(0.022)
*Household wealth index*							
First	0.011	0.0014	− 0.0025	− 0.010	− 0.024	− 0.010	0.034
	(0.031)	(0.0041)	(0.0074)	(0.028)	(0.024)	(0.011)	(0.035)
Second	− 0.070***	− 0.0241***	0.0151***	0.079***	− 0.053***	− 0.0281***	0.082***
	(0.020)	(0.0069)	(0.0048)	(0.022)	(0.019)	(0.0096)	(0.028)
Third	− 0.017	− 0.0034	0.0039	0.016	− 0.008	− 0.0030	0.011
	(0.022)	(0.0043)	(0.0050)	(0.021)	(0.020)	(0.0073)	(0.027)
*Transport*							
Vehicle or other	− 0.000	− 0.0001	0.0001	0.000	− 0.025*	− 0.0124*	0.037*
	(0.016)	(0.0049)	(0.0034)	(0.017)	(0.015)	(0.0068)	(0.021)
*General readiness*	0.0166**	0.0122**	− 0.0029**	− 0.026**	0.0229***	0.0291***	− 0.052***
	(0.0071)	(0.0050)	(0.0013)	(0.011)	(0.0061)	(0.0062)	(0.012)
*Immunization readiness*	0.0022	0.0007	− 0.0005	− 0.0024	0.0103**	0.0053**	− 0.0156**
	(0.0048)	(0.0015)	(0.0010)	(0.0053)	(0.0046)	(0.0024)	(0.0069)
*Facility type*							
Reference hospital	− 0.021	− 0.0068	0.0046	0.023	0.001	0.0004	− 0.001
	(0.016)	(0.0055)	(0.0035)	(0.018)	(0.015)	(0.0077)	(0.023)
*Facility ownership*							
Public	− 0.042**	− 0.0165**	0.0088**	0.049**	− 0.009	− 0.0055	0.015
	(0.017)	(0.0076)	(0.0035)	(0.021)	(0.015)	(0.0093)	(0.025)
Private and other	0.070***	0.0080***	− 0.0159***	− 0.062***	0.089***	0.0243***	− 0.114***
	(0.026)	(0.0028)	(0.0060)	(0.021)	(0.024)	(0.0050)	(0.027)
*Location*							
Rural/peri-urban	− 0.005	− 0.0017	0.0012	0.006	− 0.011	− 0.0061	0.018
	(0.016)	(0.0051)	(0.0035)	(0.018)	(0.015)	(0.0079)	(0.022)

* = statistically significant at *p* < 0.05

** = statistically significant at *p* < 0.01

*** = statistically significant at *p* < 0.000

In terms of predisposing characteristics, within our sample, every additional year of a mother’s education was associated with a greater rate of earlier vaccination. For instance, an additional year of education was associated with a 0.48% point increase in the probability that a mother would vaccinate her infant within 24 h of delivery and a 0.52% point decrease in the probability of never vaccinating her infant (*p* < 0.05).

At the enabling level, household wealth index scores within the second quantile (compared to the fourth, and highest, wealth quantile) were associated with a 7.0% point decrease in probability of timely uptake (*p* < 0.001) and a 7.9% point increase in the probability of never vaccinating their infant (*p* < 0.001), see Table 3.

For the external environment, higher general readiness scores were associated with timely uptake. A one-point increase in a facility’s general readiness score was associated with a 1.7% point increase in the probability of vaccination within 24 h (*p* < 0.01) and a 2.6% point decrease in the probability of never vaccinating an infant (*p* < 0.01). In addition, visiting religious-affiliated facilities compared to public facilities was significantly associated with receipt of timely BCG (4.2% point increase in vaccination by 24 h) (*p* < 0.01) and a 4.9% point decrease in never receiving it (*p* < 0.01). In addition, religious-affiliated facilities were associated with an 8% point decrease in the probability of vaccine uptake from one to seven days compared to private or other facilities (*p* < 0.001).

### Model II. Determinants of the uptake of HepB vaccine

Similar to BCG vaccine uptake, factors that affected the timely uptake of the HepB3 vaccine series varied across all three levels of the BHM.

In terms of predisposing characteristics, within the study sample, every additional year increase in a mother’s age was associated with a 0.21% point increase in the probability of an infant being vaccinated within six weeks and a 0.31% point decrease in the probability of an infant not ever being vaccinated (*p* < 0.05). Every additional year of a mother’s educational attainment was associated with a 0.77% point increase in the probability that she would vaccinate her infant within six weeks of delivery (*p* < 0.001) and a 1.2% point decrease in the probability of never vaccinating her infant (*p* < 0.001).

Within the enabling factors category, household wealth index scores within the second quantile, compared to the fourth and highest wealth quantile, were associated with a 5.3% point decrease in the probability of taking up timely vaccines (*p* < 0.001) and an 8.2% point increase in the probability of never vaccinating their infant (*p* < 0.001). Further, among the study sample, using a form of transportation other than walking to travel to the facility was associated with a 2.5% point decrease in the probability of vaccination within six weeks (*p* < 0.05) and a 3.7% point increase in no vaccination (*p* < 0.05).

At the external level, a one-point increase in a facility’s immunization readiness score was associated with a 5.3% point increase in the probability of vaccination within 14 weeks (*p* < 0.01). In addition, an additional point increase in a facility’s general readiness score was associated with a 5.2% point decrease in the probability of an infant never having been vaccinated (*p* < 0.001). Finally, visiting religious-affiliated facilities, compared to public facilities, was associated with an 8.9% point decrease in the probability of vaccine uptake at 6 weeks (*p* < 0.001).

## Discussion

In this study, we investigated the factors associated with timely infant immunization in 105 facilities in Kinshasa Province using a cohort of HIV-positive pregnant women and their infants. We observed similar immunization coverage for BCG (82.6%) and the first dose of HepB3 (68.4%) compared to the WHO/UNICEF estimates of national immunization coverage averaged from 2017 to 2020 (84.5% and 71.3%, respectively) [24].

We observed a strong positive association of factors across all three Andersen BHM categories (predisposing characteristics, enabling factors, and external environment) with timely uptake of both vaccines, BCG and HepB3. Our analyses highlight the importance of considering the effect of determinants at different levels and time intervals. The implication is that policymakers in the DRC can be more focused on implementing vaccine uptake strategies depending on their target group. For example, we identified significant determinants of timely uptake of both BCG and HepB3 at the external environmental level, focusing on intervenable facility characteristics.

Higher general readiness scores and attending a religiously affiliated facility were independently associated with both BCG and HepB3 timely uptake, a finding that aligns with previous studies highlighting vaccine storage and stockout challenges in the DRC [17, 25]. In prior work, lower general facility readiness scores have been reported as barriers to timely immunization [26]. Therefore, new vaccine introduction strategies at the facility level should prioritize contributions to general readiness, such as workforce and operations, and immunization readiness, such as reliable availability of vaccines at each facility. Ensuring adequate supply prevents stockouts, which in turn prevents unnecessary/unproductive visits to a facility. This could also bring more equity as it reduces costs for families who live further away from the facility.

In addition, visits to public facilities were negatively and independently associated with timely uptake of BCG compared with visits to religiously-affiliated facilities. In contrast, visits to private, non-religious facilities, as compared to religious facilities, were positively associated with timely uptake of both BCG and HepB3. Previous studies on immunization uptake in SSA have cited a facility’s religious affiliation driving a mother’s choice to seek care there [17]. In terms of private facilities, studies show that mothers visiting private facilities tend to be of higher income levels, [27] a factor that was positively associated with timely vaccine uptake and may explain our findings. Future research could examine the reasons that vaccination rates are higher among those who select private facilities.

Within the category of predisposing characteristics, we found that the mother's educational attainment and age at the time of enrollment were positively associated with the timely uptake of vaccines, which indicated that knowledge, awareness, and experience with preventative care were vital for timely vaccine uptake. A mother's education level is an established predictor of infant immunization in low- and middle-income countries [19, 21, 28, 29] and education is a valuable solution to overcome challenges to vaccine uptake [30, 31]. Mothers primarily receive healthcare information from two sources: health workers during ANC visits, and their families and communities [17, 32]. Previous studies have shown that knowledge about HBV risk and the vaccines’ protection is low among Congolese individuals, [17, 33] with one study finding a basic knowledge of HBV among only 33.2% of healthcare workers [33] and another finding that only 31.2% (87/280) of pregnant women knew how HBV was transmitted [34]. To enhance a mother’s understanding, efforts to promote vaccine knowledge should be directed at her two main channels of information, healthcare facilities during ANC visits and her local community. In terms of healthcare facilities, training programs that include vaccine information should not only involve vaccination personnel, but also ANC staff responsible for conveying information to expectant women [17]. At the community level, approaches to overcome knowledge barriers should encompass the entire social network of the mother, including family and friends [33].

Household wealth status was positively associated with timely BCG vaccination, consistent with other studies [19, 35, 36]. A possible explanation for this observation is the cost of the vaccinations. Although vaccinations are technically free in the DRC, facilities often require vaccination fees for a vaccine card and well-baby consultation. In addition, indirect costs such as transportation and income loss may act as economic burdens obstructing vaccine uptake [19, 37]. The economic burden was further substantiated by our finding that mothers were more likely to vaccinate their infants on time, or ever, if they lived within walking distance to the facility. This finding was confirmed by earlier studies in SSA that distance to facility, travel time, and need for transport were negatively associated with immunization uptake [19, 28]; one such study found that traveling a distance of over 30 min by foot compared to a shorter distance reduced vaccine uptake by one-third [28]. These implications for the enabling factors require policy intervention to reduce the economic burden of infant vaccines, such as transparency and standardization of vaccine costs across facilities and incentives for mothers living beyond walking distance from facilities.

A significant strength of this study was the aggregation of facility-level and individual-level longitudinal data across many facilities. Few studies have looked at a combination of the individual-level determinants of the mother-infant pair and the facility-level determinants of vaccine uptake. The study employs a unique approach to controlling for confounding by using data from both the supply (environment) and demand (mother-infant pair) side. This study's access to longitudinal panel data of over 2,000 women and inventory data about each of the study facilities allowed us to evaluate a comprehensive list of determinants across the BHM levels that determine timely uptake of vaccines. Beyond the unique challenge of administering the vaccine within 24 h of birth, these determinants highlight the need for an implementation strategy to be rolled out alongside universal HepB-BD. Previous studies demonstrate that timely uptake remains low in countries that have previously adopted HepB-BD because there is no clear guidance to overcome individual- and facility-level challenges [38, 39]. Our study's main policy implication was to highlight the barriers to current BD vaccines—and HepB3 vaccine—in a context that strives to include the HepB-BD vaccine in its national immunization schedule. Policymakers may use these findings as evidence when developing a future implementation strategy streamlining all three BD vaccines—HepB-BD, BCG, and OPV0—within the first 24 h of life. Findings from this study can help national, sub-national, and facility-level stakeholders to strengthen the uptake of both BD vaccines and other available vaccines for infants across the DRC and SSA.

Despite the study's strengths, it was not without limitations. Our assessment leveraged sample participants from a cohort of women already enrolled in an HIV continuous quality improvement study, which impacted the generalizability of this study. However, vaccine uptake in this population was similar to the national average, and factors influencing vaccine uptake were aligned with other infant immunization studies in the Congo and elsewhere in SSA [19, 28–31, 35, 36, 40]. In addition, previous and ongoing studies conducted in the same study clinics observed similar proportions of participants lost to follow up [21, 41]. The measure of the outcome variables, vaccine uptake status and timing, may have suffered from recall bias because of the contemporaneous approach of capturing vaccine dates during study interviews. The study staff reviewed infants' vaccine records to correct any errors in logging the vaccine dates to help alleviate any errors. Another limitation of this study is that estimates are not causal but rather represent associations. In addition, only mothers recruited pre-delivery (approximately half of the sample) responded to a question regarding how many ANC visits they had attended because study staff dropped the question among mothers recruited post-delivery. Finally, while the parent study did experience significant rates of LTFU, study staff followed up with respondents to understand reasons for LTFU and recapture some of the data otherwise lost. We were able to weigh LTFU within the 'never vaccinated' rate to represent more accurate rates of failure to vaccinate.

## Conclusions

Our findings reveal the factors that most influence timely vaccine uptake of BCG and HepB3 among a cohort of mother-infant pairs across 105 facilities in the Kinshasa Province. We found that higher educational attainment, age, and level of wealth among mothers were positively associated with timely vaccination. In addition, a mother’s proximity/ability to walk to a facility and her choice of facility impacted the status of her infant's vaccine uptake. A mother visiting a facility with higher general and immunization readiness and religious affiliation or private-ownership led to a higher probability of timely infant immunization. Policymakers can use these findings to develop implementation guidance to ameliorate the timely delivery of current BD vaccines and to anticipate potential factors that may impact the future distribution of HepB-BD in the DRC.

## Data Availability

The datasets used and/or analysed during the current study are available from the corresponding author on reasonable request.

## Abbreviations

AIC: Akaike’s information criterion
ANC: Antenatal care
BCG: Bacille Calmette–Guérin vaccine
BD: Birth-dose
BHM: Andersen behavioral health model
CQI: Continuous quality improvement
DRC: The Democratic Republic of the Congo
HBsAg: Hepatitis B surface antigen
HBV: Hepatitis B virus
HepB-BD: Hepatitis B birth-dose vaccine
HepB3: Hepatitis B vaccine series
HIV: Human immunodeficiency virus
IQR: Interquartile range
LTFU: Loss-to-follow-up
MTCT: Mother-to-child-transmission
OPV0: Oral polio vaccine
SARA: WHO service availability and readiness assessment
S.D.: Standard deviation
SSA: Sub-Saharan Africa
UNICEF: United Nations Children’s Fund
WHO: World Health Organization
